# Fine scale mapping of water sources in low-income settings: A comparative study in Misungwi, Tanzania

**DOI:** 10.1371/journal.pone.0319603

**Published:** 2025-03-21

**Authors:** Claudia Duguay, Charles Thickstun, Jacklin F. Mosha, Tatu Aziz, Alphaxard Manjurano, Alison Krentel, Natacha Protopopoff, Manisha A. Kulkarni

**Affiliations:** 1 School of Epidemiology and Public Health, University of Ottawa, Ottawa, Canada,; 2 National Institute for Medical Research Tanzania, Mwanza Research Centre, Mwanza, Tanzania,; 3 Bruyère Research Institute, Ottawa, Ontario, Canada,; 4 Faculty of Infectious and Tropical Diseases, Disease Control Department, London School of Hygiene and Tropical Medicine, London, United Kingdom; Gadjah Mada University Faculty of Medicine, Public Health, and Nursing: Universitas Gadjah Mada Fakultas Kedokteran Kesehatan Masyarakat dan Keperawatan, INDONESIA

## Abstract

Access to safe water, sanitation, and hygiene is a basic human need for health and well-being. Yet, 2.2 billion people globally in 2022 did not have access to safely managed drinking water. Presently there are no publicly available methods for monitoring and measuring access to water sources in low-income settings at a fine spatial scale. The objective of this study was to map and identify areas with improved and unimproved water points in Misungwi, Tanzania using two different methods: 1) community mapping with direct field observations, and 2) drone imagery. We quantified and summarized the number of improved and unimproved water sources, as defined by the WHO/UNICEF Joint Monitoring Programme core questions and noted their specific uses where applicable. We also compared the results of both data collection methods outlining their respective advantages and limitations. The community maps and direct field observations not only served as a method to identify water sources, but also provided insights into how community members used and interacted with each water source. In contrast, the drone imagery only served as a method to systematically identify water sources in the study area. A notable advantage of the drone imagery, however, was its ability to identify more unimproved water sources (225 vs 90) compared to the direct field observations. Both methods were effective in identifying water sources at a fine scale, but the drone imagery involved a more time-intensive process, demanded advanced skills, and incurred a higher cost compared to the community mapping with direct field observations. This study highlights the need for accurate and readily accessible data on water sources which is imperative for planning, developing, and managing improved water sources, especially in underserved areas such as Misungwi, Tanzania.

## Introduction

Access to safe water, sanitation, and hygiene (WASH) is a basic human need for health and well-being [1]. Yet, 2.2 billion people globally in 2022 did not have access to safely managed drinking water [1]. Access to safe water is targeted by the Sustainable Development Goals (SDG), specifically SDG 6 on clean water and sanitation for all [2].The SDGs are a group of 17 interrelated goals set by the United Nations for 2030 that provide a global framework to address global challenges such as poverty, hunger, diseases, and discrimination against women and girls [3]. The SDGs are interrelated, where progress in one goal will affect the outcomes of others. It is evident that SDG 6 cannot be achieved without addressing other essential SDGs including poverty reduction (SDG 1), food security (SDG 2), improving population health (SDG 3), education (SDG 4), and peace and human rights (SDG 16) [1,4–8]. For example, SDG 6.1.1 aims to increase the proportion of the population using safely managed drinking-water services, and SDG 3.9.2 aims to substantially reduce the number of deaths related to unsafe water [1,8].

The World Health Organization and UNICEF collaborated to form the Joint Monitoring Programme (JMP) for Water Supply, Sanitation and Hygiene that is responsible for monitoring the SDG 6 targets through core questions for WASH indicators [9]. There are six core questions for drinking water that are categorized into five-levels, which are coined as a “service ladder”, and are based on the source and the time it takes to retrieve the water [9]. The five different levels include: 1) surface water, defined as retrieving drinking water directly from a river, dam, lake, pond, stream or canal; 2) unimproved, defined as retrieving drinking water from an unprotected dug well or unprotected spring; 3) limited, defined as retrieving drinking water from an improved source but collection time exceeds 30 minutes for a round trip; 4) basic, defined as retrieving drinking water from an improved source and collection time does not exceed 30 minutes for a round trip; 5) safely managed, defined as retrieving drinking water from an improved water source that is located on premise, available when needed and free from contamination [9]. These levels can be further dichotomized into unimproved or improved drinking water where unimproved drinking water sources include surface and unimproved water, and improved drinking water sources include limited, basic and safely managed water [9].

Although the WHO/UNICEF JMP only has indicators for drinking water, WASH-attributable diseases can be spread from a range of transmission routes including ingestion, contact with contaminated water, or through vectors that need water to complete their lifecycle [10]. In fact, 2.5% of all deaths globally in 2019 were from four diseases that are spread through unsafe WASH practices including diarrhoea, acute respiratory infections, undernutrition, and soil-transmitted helminthiases [11]. However, this is likely an underestimation since there are other WASH-attributable diseases including dengue, Japanese encephalitis, lymphatic filariasis, malaria, onchocerciasis, and schistosomiasis [11,12].

Despite the global burden of WASH-attributable diseases and the global targets set by the SDGs, there are no publicly available data for monitoring and measuring access to unimproved water for drinking and other domestic uses in low-income settings at a fine spatial scale. In research, water availability is typically represented by proxies such as rainfall or vegetation indices like the normalized difference vegetation index (NDVI) [13,14]. Gridded raster data on NDVI globally are publicly available through the Moderate Resolution Imaging Spectroradiometer (MODIS) at a spatial and temporal resolution of 250 m and 16-days, respectively [15]. NDVI can identify large unimproved water sources such as unprotected springs and ponds (NDVI < 0) [16,17], but it cannot identify unprotected wells which have an average diameter of 1.2 meters [18]. There are also publicly available nationally representative census data through the Demographic Health Survey (DHS) used to monitor and evaluate progress towards multiple SDGs, including SDG 6. The DHS uses the core questions outlined by the WHO/UNICEF JMP including the type of drinking and nondrinking water used by each household [19]. Although these surveys are nationally representative, it does not allow for fine spatial analyses. For instance, in a singular region of Tanzania (Mwanza), the point density for the number of households sampled per square kilometer was 0.00098 (22 points to cover 22,233 km^2^) in 2022 [19]. The WHO/UNICEF JMP also reports yearly national metrics for the proportion of people using unimproved drinking water, but this is a single metric without any spatial component [20]. Finally, there are publicly available repositories for the location of water points and their status managed by Water Point Data Exchange [21], however, the availability of these data points are scarce – for instance, in Tanzania, there is only data for 10 of the 184 districts (5%) [21].

Fine spatial scale data is needed to accurately capture water availability in low-income settings where inequities result in higher vulnerability to WASH-attributable diseases. There is a particular need for data on unimproved water sources such as surface water (river, stream, lake, pond), unprotected dug wells, and unprotected springs which can inform and provide stakeholders with information of geographic hotspot areas with unimproved water points. The objective of this study was to map and identify areas with improved and unimproved water points in Misungwi, Tanzania, using two different methods: community mapping with field observations, and drone imagery.

## Methods

### Study setting

**Fig 1:**
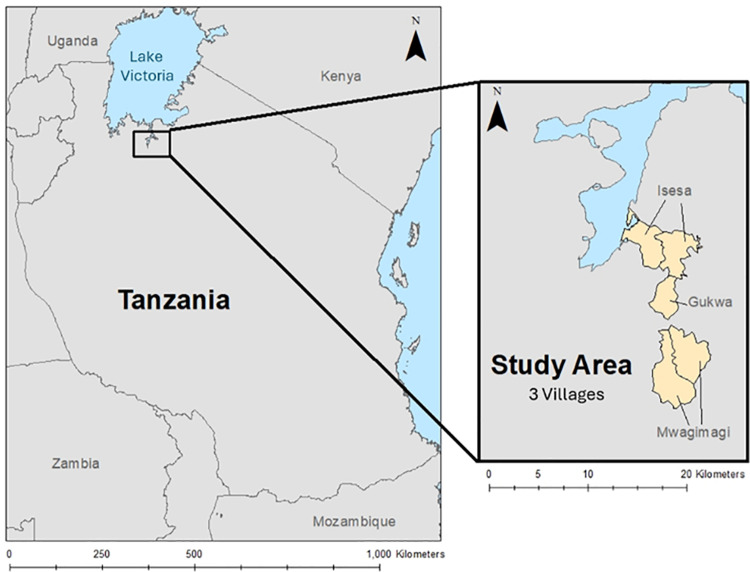
Map highlighting the study area in reference to Lake Victoria, and a detailed view of the 3 villages that make up the study area. Map content was produced with Esri ArcGIS software using study data and data provided by GADM and Natural Earth available online: https://gadm.org/download_country.html and https://www.naturalearthdata.com/.

This study was nested in a four-arm, single blinded, parallel, cluster randomized control trial assessing the efficacy of three dual active-ingredients long lasting insecticidal nets for the control of malaria in Misungwi, Tanzania (Fig 1) [22]. Misungwi has two rainy seasons from March to May (Masika – long rains), and from October to January (Vuli – short rains). This study specifically used data from three (Gukwa, Mwagimagi and Isesa) of the 78 villages from the main trial, which were selected based on high infection status for malaria and schistosomiasis (two WASH-attributable diseases) and proximity to Lake Victoria [22,23]. A recent survey conducted in these villages in 2022 found that no households (n =  92, 100%) in these three villages used improved drinking water sources [23], with the most fetching water from unprotected springs (83%), followed by surface water (12%), and unprotected wells (5%).

### Data sources

#### Community maps and direct field observations.

This study used two different methods to identify water sources in the three villages including community mapping with field observations, and drone imagery. Participative community mapping activities and direct field observations were conducted in August 2022. Data collection methods are described in detail elsewhere [23]. Briefly, community maps were created by village members to identify pre-defined features in their village (i.e., roads, water sources and their specific uses, schools, health centres) to have a better understanding of the different assets in each village [24]. Specifically, ten community members above the age of 18 years from each village, with at least 5 men and 5 women per village, were selected by village leaders to create a map of their village with one map being create by a group of men, and one map created by a group of women.

The direct field observations were characterized by an opportunistic methodology, informed by the community maps (i.e., using different assets such as roads and health centres as reference points) and informal interviews by community members [25]. We also identified other water sources that were not identified in the community map to understand water access for drinking and other domestic uses. We continued to identify water sources until no new locations were being identified or provided by community members. While this process was not conducted using a systematic methodology, it allowed us to capture the relevant water sources recognized and utilized by the community. Geographic coordinates and pictures were collected for all water sources in the study area using handheld GPS devices and an iPhone 13, respectively. If people were present at the water source, informal interviews were conducted to inquire about specific water uses (i.e., for drinking, for bathing, visit frequency). We then quantified and summarized the number of improved and unimproved water sources, as defined by the WHO/UNICEF JMP core questions, and noted their specific uses where applicable [9]. Finally, we determined the number of households (retrieved from the census data from the main trial) that have access to improved water sources within a 30-minute round which the WHO/UNICEF JMP classifies as “basic” access to water [9]. This calculation was implemented in ArcMap version 10.8.1 (ESRI, Redlands, CA, USA) and included households within a 1 km (15-min) buffer around each improved water source, using an average walking speed of 1.2 m/s [26]. However, it is important to acknowledge that walking speeds can vary among individuals, and households may not necessarily utilize the nearest water source. Despite these limitations, and without directly surveying and timing individuals, this approach represents the most accurate assessment to identify areas with basic and limited access to water for this study.

#### Drone imagery.

The drone imagery was captured in September 2022. We worked with Notice Kilimanjaro, an experienced and authorized Tanzanian drone operating media company (TCAA authorized and licensed to carry out drone flights across Tanzania) to map the three villages using a Mavic 2 Pro quadcopter drone. The drone was flown using parallel flight lines (a lawn-mower flight pattern) at an altitude of 100 m, giving a ground-sampling distances of 3 cm, and capturing approximately 2 km^2^ a day. This allowed for a cumulative coverage of 64 km² over 25 days, representing the total area of all three villages. Notice Kilimanjaro processed all drone images prior to analysis. Manual delineation (points) of the centroid and the diameter of all water sources of the georeferenced images were done using ArcMap version 10.8.1 (ESRI, Redlands, CA, USA). We then quantified and summarized the number of improved and unimproved water sources based on visual appearance from the georeferenced image[9 ]. Descriptive statistics were used to summarize the number and type of water sources, dichotomized as either improved and unimproved, using frequencies and proportions. As with the community walkthrough analysis, we determined the number of households with “basic” access to water by calculating the number of households that have access to improved water sources within a 30-minute round (1 km buffer around each improved water source) [9].

### Comparative analysis

A comprehensive qualitative analysis comparing the results of each data collection method and outlining their respective advantages and limitations was conducted. Specifically, we identified key themes that emerged from each approach, including their ability to identify the different water sources used by community members in all three villages, and the financial and time resources required for each method.

### Ethics statement

The protocol for this study was reviewed and approved by the institutional review boards, of the University of Ottawa (Canada) and Medical Research Coordinating Committee of the National Institute for Medical Research (Tanzania). Written informed consent forms were obtained from all participants prior to data collection activities. Additional information regarding the ethical, cultural, and scientific considerations specific to inclusivity in global research is included in the Supporting Information (S1 File: Inclusivity in Global Research)

## Results

### Summary of results

#### Community maps and direct field observations.

Six community maps were created in the three selected villages (all six maps are published in [23]). Community maps primarily identified unimproved water sources as well as other assets including schools, health centres and Village Executive Officer offices. Only one water source in all community maps met the criteria for an improved source of water, which was a public tap identified by the women in Isesa. All other sources of water could be classified as unimproved surface water (i.e., wells, ponds, river, lakes).

**Fig 2:**
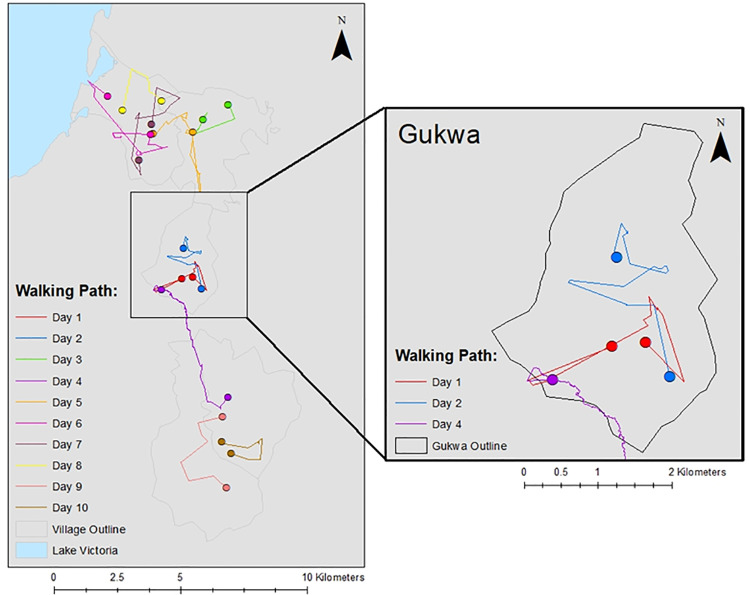
Map of the study area outlining the daily walking paths for the community walkthrough. Each path was informed by the community maps and informal interviews with community members. Map content was produced with Esri ArcGIS software using study data and data provided by GADM and Natural Earth available online: https://gadm.org/download_country.html and https://www.naturalearthdata.com/.

**Fig 3:**
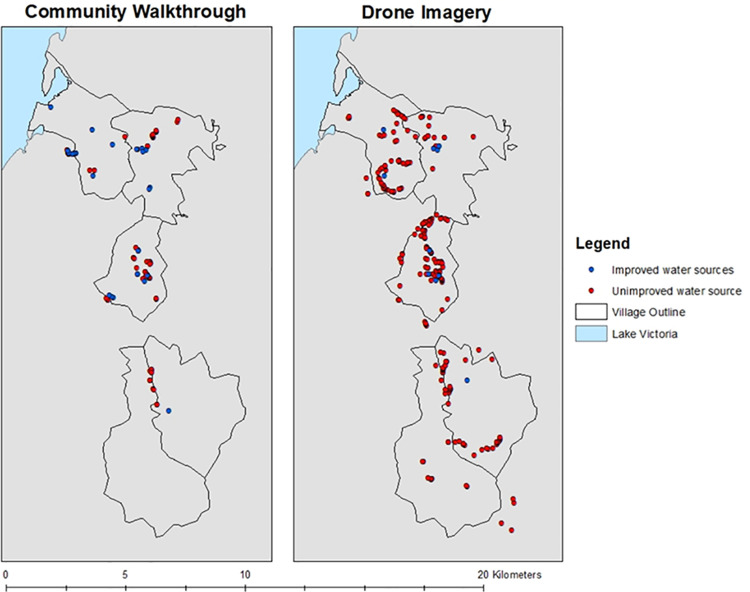
Map of the study area outlining the location of improved (blue), and unimproved (red) water sources identified in the community walkthrough and the drone imagery. Map content was produced with Esri ArcGIS software using study data and data provided by GADM and Natural Earth available online: https://gadm.org/download_country.html and https://www.naturalearthdata.com/.

The median daily walking distance for the direct field observation was 5.7 km for a total of 10 days, with a minimum daily walking distance of 3.6 km and a maximum of 9.7 km (Fig 2). A total of 114 water sources were identified during the direct field observations (Fig 3), with the majority (n = 90, 79%) being unimproved water sources (i.e., surface water, unprotected wells) (Table 1).

**Table 1:** Characteristics of water sources identified in the community maps and direct field observations, and the drone imagery.

	Total number of water sources	Total number of unimproved^*^ water sources	Total number of improved^#^ water sources
Direct field observations	114	90 (79%)	24 (21%)
Drone imagery	236	225 (95%)	11 (5%)

* Unimproved water sources include surface water, unprotected springs, and unprotected wells [9].

# Improved water sources include public taps, boreholes, and protected wells [9].

**Fig 4:**
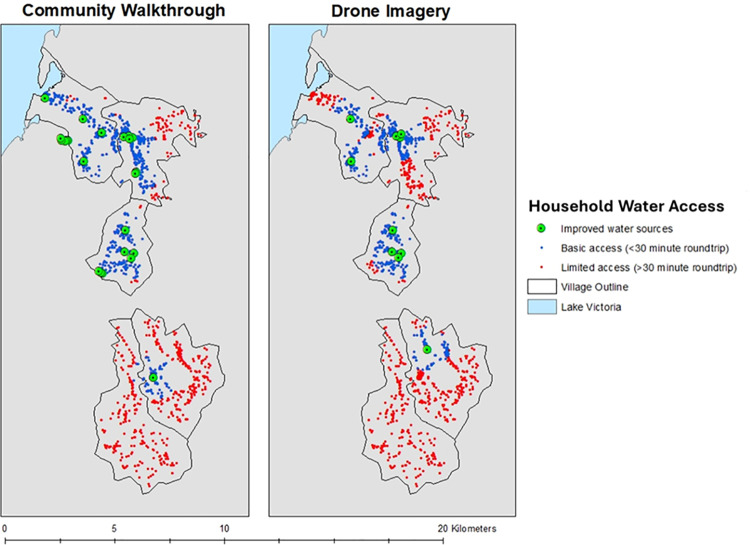
Map of the study area outlining households that could be served by an improved water source within a 30-minute round trip (within a 1km buffer of an improved water source). Map content was produced with Esri ArcGIS software using study data and data provided by GADM and Natural Earth available online: https://gadm.org/download_country.html and https://www.naturalearthdata.com/.

A total of 1459 households in the study area were identified in the census data, of which nearly two thirds (n = 921, 63%) had access to basic water (households with access to an improved source within a 30-minute round trip) (Fig 4). The median distance to the nearest improved water source identified in the community walkthrough was 630 m with a minimum and maximum distance of 6 m and 4938 m, respectively.

#### Drone imagery.

**Fig 5:**
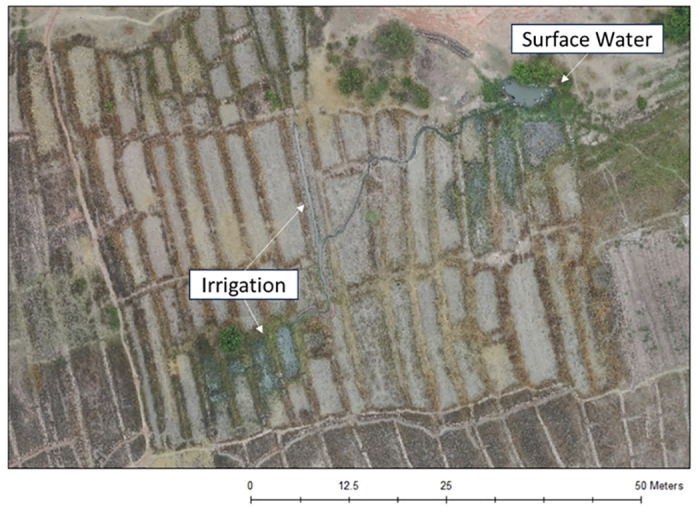
Example of the difference between surface water and water suspected to be used for irrigation in Gukwa.

A total of 236 water sources were identified in the drone imagery (Fig 3), with the majority (n = 225, 95%) being unimproved water sources (i.e., surface water, unprotected wells), though less improved water sources were identified in the drone imagery compared to the direct field observations (11 compared to 24) (Table 1). Based on the shape of the surface water surrounding and bordering crop lines, 47 of the 236 (20%) water sources were suspected to be used for farming and irrigation as seen in Fig 5, and not for human consumption and use. The median diameter of all the unimproved water sources was 5.4 m, with a minimum diameter of 0.3 m and a maximum of 45.8 m.

Nearly half (n = 649, 44%) of the households in the study area identified in the census had basic access to water (households with access to an improved source within a 30-minute round trip) (Fig 4). The median distance to the nearest improved water source identified in the drone imagery was 1114 m with a minimum and maximum distance of 10 m and 6245 m, respectively.

### Comparative analysis

Integrating the two methods provided a comprehensive overview of water access for drinking and domestic uses in the study area. Nevertheless, it is imperative to consider the respective advantages and disadvantages associated with each individual method (Table 2).

**Table 2:** Characteristics and resource requirements for data collection using community maps and direct field observations, and drone imagery to identify water sources at a fine scale in Misungwi, Tanzania. Advantages and disadvantages are indicated for each characteristic.

Characteristics/resources	Community maps and direct field observation	Drone imagery
Information derived	Advantage • Contextualized water uses and behaviour. • Can assess usability, functionality, and quality of improved water sources (i.e., borehole). • GPS location of water source. • Better at identifying improved water sources compared to drone imagery. Disadvantage • Study data is constrained to a specific set of objectives (i.e., identification of domestic and drinking water sources).	Advantage • Systematically identified all available water sources in the study area^*^. • Could detect water sources as small as 30 cm in diameter. • Drone imagery data could be used for other studies with varying objectives (i.e., crop classification, urban planning, disaster response). Disadvantage • Could not delineate if water was used by humans and in what capacity (i.e., drinking, domestic use) • Could not assess water source functionality and quality.
Financial cost	• Field personnel and travel: $ USD 2,500	• Service, field personnel, and travel $ USD 8,000
Time commitment	• 13 days for data collection • 7 h for data analysis	• 25 days for data collection • 80 h for data analysis

* Based on previous survey results [23], no household in this study area had access to improved water sources, indicating that piped water and rainwater collection was not an important consideration.

### Information derived

All methods collected water use information at a fine scale including the GPS location of water sources for the direct field observations and a 3 cm resolution for the drone imagery. However, one of the fundamental differences in the methods lies in the types of information obtained from each method.

The community maps and direct field observations contextualized and provided an indication of how the community members in all three villages used and interacted with surface water (i.e., used for domestic use, used for farming and other domestic uses, reserved for cattle). A contextual understanding of water source uses and behaviours are described in detail elsewhere [23]; but briefly, we found that children primarily fetched water, and that although ponds and wells were sourced from the same body of water (separated by an arbitrary border), unprotected wells were dedicated for drinking water and ponds were dedicated for other domestic uses and cattle. It was also evident from the community walkthrough that although improved water sources were identified (e.g., hand pumps sourced from wells or boreholes), they were not in use because of unpleasant taste and smell. This aspect was especially pertinent when analyzing the drone imagery conducted one month later in September 2022.

**Fig 6:**
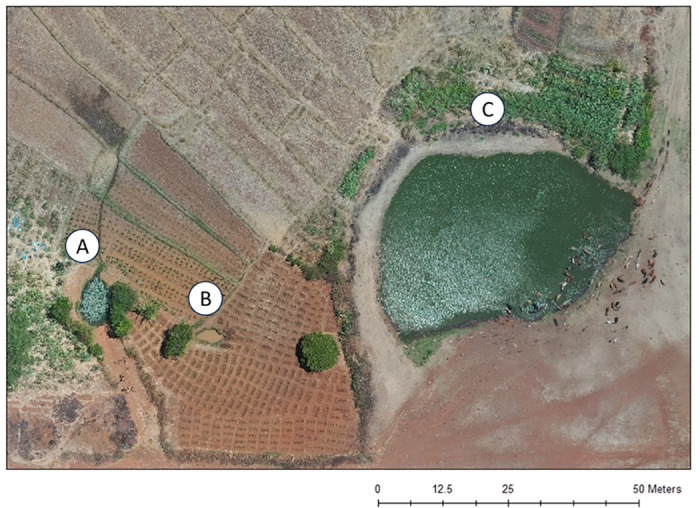
Water sources identified in the drone imagery in Mwagimagi compared to the contextual information gathered during the community walkthrough including a water source used by humans for all domestic uses (A), a water source that was not found in the community walkthrough (B), and a water source reserved for cattle (C).

The drone imagery identified 122 more water sources (107% more water sources) than the field observations, specifically more unimproved water sources, but the absence of contextual details posed a challenge in determining their relevance for human use and disease risk. For example, in Mwagimagi, the drone imagery identified three water sources of varying sizes (Fig 6A–6C) with the evidence of cattle inside and surrounding one of the three water sources (Fig 6C). During the direct field observations, we identified two of these three water sources (Fig 6A and 6C) that were identified in the drone imagery; walking past the second water source (Fig 6B) even though it was near the other two water sources (Fig 6A and 6C). Additionally, community members revealed that the first water source (Fig 6A) was used by humans for all domestic uses including drinking water, and that the third water source (Fig 6C) was reserved for cattle and was not used by humans because it was considered “dirty”. This type of information is invaluable, particularly as the presence of cattle has implications for certain water-borne diseases (i.e., cryptosporidium, hybrid species of schistosomiasis [27,28]).

**Fig 7:**
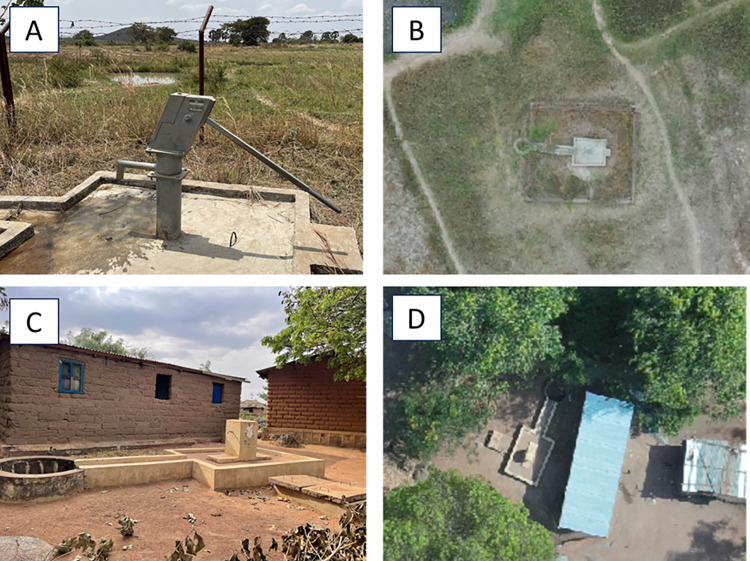
Comparing a functional borehole identified in the direct field observations (A) and drone imagery (B) in Gukwa and a non-functional borehole identified in the direct field observations (C) and drone imagery (D) in Isesa.

Another prominent difference between the two methods was the ability to assess the usability, functionality, and quality of the water sources (although not performed exhaustively or systematically in this study), which was only possible with the direct field observations. For example, we identified and assessed the functionality of a borehole in Gukwa (Fig 7A) and Isesa (Fig 7C) which we later identified in the drone imagery (Fig 7B and 7D). The ability to note the functionality and quality of water proved to be a significant advantage in this study area as many boreholes were not functional, e.g., such as the one in Isesa (Fig 7C and 7D). The community walkthrough also highlighted that the availability of improved water sources did not imply the utilization of that water source, where community members noted that they did not use the improved water sources for drinking or other domestic uses because of unpleasant taste and smell. Therefore, although we analyzed the number of households that could be served by “basic” water sources, those metrics should be interpreted with caution given the information derived from the community walkthrough.

### Financial cost

The second notable difference between the two methods pertained to the project budget and costs. This study was nested in a randomized control trial assessing the efficacy of next-generation long lasting insecticidal nets for the control of malaria [22]. Therefore, the cost of the two methods reflects the leveraging of available resources (i.e., infrastructure, staff, participant recruitment, survey questionnaire) and established in-country partnerships. The cost for the drone imagery was significantly more than the community mapping with field observations (USD$ 2,500 compared to USD$ 8,000), which represent the cost for the field personnel, travel, and service. The community maps and direct field observations were conducted by three people including a local field personnel and two Canadian (international) doctoral students for 15 days whereas the drone service included a drone pilot, a driver, and the drone image processing for 30 days.

### Time commitment

The third notable difference between the two methods pertained to the time commitment required for both data collection and analysis. Although less labour intensive than the community walkthrough, the drone imagery took 12 additional days for data collection and 73 additional hours for data analysis, not including the time for image processing prior to analysis, compared to the community mapping with field observations. The community mapping with field observations, and the drone imagery were collected by research assistants and a drone service, respectively, however, the research assistant responsible for the data analysis was present for both data collection activities, although not necessary for the drone service. The extent of the direct field observations analysis included stratifying the results by each village and to dichotomize and summarize the different water sources using frequencies and proportions. The drone imagery was complex and required a substantial time commitment and skills to analyze. Most of analysis time was spent systematically identifying water sources within a study area 64 km^2^ (≈50 h). The remaining time was spent processing and uploading the images, which amounted to 1 terabyte of data.

## Discussion

Fine spatial scale data is imperative to capture information on water availability in low-income settings where inequities result in higher vulnerability to WASH-attributable diseases. This study focused on three villages in Tanzania with a history of using unimproved water sources for drinking and other domestic uses. We identified water bodies, including surface water and unprotected wells, ranging from 30 cm to 46 m in diameter using two methods. The community maps and direct field observations identified, contextualized, and provided an understanding of how community members in all three villages used and interacted with water sources, while the drone imagery systematically identified water sources in the study area, including more unimproved water sources (225 vs 90) and less improved water sources (11 vs 24) compared to the direct field observations. Both methods were effective in identifying water sources at a fine scale, but the drone imagery involved a more time-intensive process, demanded advanced skills, and incurred a higher cost compared to the community mapping with direct field observations.

It is evident from this study that publicly available environmental data or nationally representative data such as the DHS fail to capture the heterogeneity of risk factors for WASH-attributable diseases and access and usage of improved and unimproved water sources at a fine spatial scale (i.e., at an individual or household-level). For instance, in this study, the median size of water sources identified in the drone imagery was 5.4 m, and the direct field observations noted that community members use these water sources for drinking and other domestic uses, potentially exposing them to WASH-attributable disease. While there is no single proxy for water availability or environmental risk factors for WASH-attributable diseases, studies typically use publicly available proxy environmental data at spatial resolutions of 250 m (i.e., NDVI [29]) or 1 km (rainfall [30]) as risk factors for diseases [31–33] and to even to predict disease prevalence to inform control programs [34].

As climate change is expected to alter the patterns and intensity of rainfall, temperature, and other environmental factors, it will likely influence the distribution and prevalence of WASH-attributable diseases [35–37]. It is evident that WASH-attributable diseases exhibit considerable temporal and spatial heterogeneity, making it essential to monitor and identify risk areas at a fine scale for effective disease prevention and control. Direct field observations and drone imagery are effective methods for identifying fine scale features in the environment that are beyond the scope of publicly available methods and can expand beyond the application of identifying water sources in low-income settings. Drone imagery has proven to be a valuable tool in crop classification for agriculture [38], search and rescue missions to identify humans after a natural disaster [39], and to aid in study designs to identify households where census data is either incomplete or not available [40,41].

### Limitations

This study has some limitations to be considered. We could not comprehensively compare the water sources identified in the direct field observations and the drone imagery for two reasons: 1) the presence of trees obstructing the aerial view necessary for capturing all water sourced during the drone imagery process, and; 2) we could not confidently discern which water source in the direct field observation corresponded to which water source identified in the drone imagery, likely because of limitation in the accuracy of the GPS handheld device. Typically, handheld GPS devices are accurate within a range of 5 to 10 m [42]. Therefore, when there was the presence of multiple water sources in the drone imagery that did not correspond to the number of water sources identified in the direct field observation, we could not discern which water source from the direct field observation corresponded to the drone imagery. This limitation posed challenges in a direct one-to-one comparison between the points obtained from the direct field observations with those captured in the drone imagery.

Another limitation is that both methods collected data at one point in time during the dry season, limiting the ability to evaluate fluctuations in water availability that occur seasonally. Specifically, in this study area, surface water and unprotected wells can evaporate completely during the dry season [32] and behaviours in accessing and using unimproved water sources also vary with the varying availability of water across the seasons [23]. Although this study did not assess fluctuations in water availability, it is evident that the study area faces significant challenges related to safe water infrastructure and usage which was identified by both the direct field observation and drone imagery. Conducting similar research across the different seasons is recommended, especially given the expected shifts in rain patterns due to climate change.

## Conclusion

Access to safe water is a basic human need for health and well-being [1] and yet current methods for monitoring and measuring access to improved and unimproved water for drinking and other domestic uses in low-income settings are not sufficient at a relevant scale. Accurate and readily accessible data on water sources is crucial for various stakeholders, including governments, researchers, international organizations, and non-governmental organizations. This information is imperative for planning, developing, and managing improved water sources, especially in underserved areas such as Misungwi, Tanzania. The importance of reliable data becomes particularly evident when striving to achieve the SDGs set for 2030 [3]. Such data serves as a foundation for informed decision-making and targeted efforts to ensure the provision of safe and sustainable water, addressing a pressing global need.

## Supporting information

S1 File Inclusivity in Global Research (DOCX)
